# Whole grain intake remains unchanged in the UK, 2008/2012–2016/2019

**DOI:** 10.1017/S0007114525104091

**Published:** 2025-08-14

**Authors:** Inga Kutepova, Colin D. Rehm, Samara Joy Smith

**Affiliations:** 1 Life Sciences, PepsiCo R&D, Reading, UK; 2 Life Sciences, PepsiCo R&D, Purchase, NY, USA

**Keywords:** Whole grains, National Diet and Nutrition Survey, UK

## Abstract

To address limited data on whole grain (WG) consumption in the UK, we investigated trends and socio-demographic patterns of WG consumption from the National Diet and Nutrition Survey from 2008/2012 to 2016/2019 and examined the relationship between WG and dietary intakes. We analysed 15 655 individuals aged ≥ 1·5 years who completed a 4-day food diary. WG consumption was quantified by estimating the WG content of individual foods using publicly available ingredient information. Survey-weighted mean WG consumption over time and by population sub-group was calculated. Survey-weighted trend tests and Wald tests were used. Total WG intake in the general population did not change from 2008/2012 to 2016/2019. WG from high-fibre cereals and bread declined by 16·2 % (11·1–9·3 g/d) and 19·4 % (12·4–10 g/d), respectively, while other cereals (e.g. rice/pasta) increased by 72·5 % (4·0–6·9 g/d), but contributed considerably less than other categories. In the most recent data (2016–2019), older adults (65+ years) had the highest energy-adjusted WG consumption, followed by children 1·5–3 years. Individuals with lower incomes, adolescents and current smokers consumed the least WG. Whole grain intake was associated with generally higher quality diets, specifically consuming more fibre, potassium, Ca, Fe, Mg, fruits/vegetables, pulses/nuts and oily fish and consuming less free sugars, total fat, saturated fat, Na and red/processed meat (*P*_trend_ < 0·001 for all). Despite some dietary improvements in the UK, WG intake appears unchanged from 2008/2012 to 2016/2019.

Whole grains (WG) retain bran, endosperm and germ, which are rich in fibre, vitamins, minerals, lignans and phytochemicals. WG offer a more nutrient-dense option compared with refined grains that lose these vital elements during processing^(1)^. The Global Burden of Disease study underscores the potential impact of low WG consumption, estimating nearly 376 00 avoidable deaths across Europe in 2021, along with over 8·1 million Disability-Adjusted Life Years, mostly due to CVD^(2)^. Whole grains consumption is associated with decreased risk of all-cause mortality, type 2 diabetes, CVD, cancer and obesity^(1,3–8)^. While fibre found in WG contributes to the protective effects against CVD, the association persists even after adjusting for fibre intake, suggesting other active components of WG may be involved potentially phytochemicals and/or micronutrients^(9)^.

In the UK, recent data on WG intake are limited. The most recent analysis used 2008–2014 National Diet and Nutrition Survey (NDNS). The data from 2008 to 2011 showed that over 70 % of UK individuals failed to meet recommended WG intake levels (by some European countries, the UK does not currently have a formal quantitative recommendation)^(10,11)^. Furthermore, approximately one in five individuals did not consume any WG^(10–16)^. The UK Eatwell Guide encourages ‘choosing wholegrain or higher fibre versions where possible’, but does not provide an explicit recommended WG intake amount^(17)^. This study assessed WG consumption in the UK using data from the NDNS rolling programme between 2008/2012 and 2016/2019. Since the NDNS data does not include a WG variable, a variable was created for WG consumption, and publicly available ingredient data were leveraged.

## Methods

### Data source and population

NDNS (2008–2019) is an ongoing cross-sectional survey representative of the population across the UK, including England, Scotland, Wales and Northern Ireland^(18)^. This data provide a comprehensive overview of dietary habits and nutrient intake across various demographic groups.

### Dietary assessment methodology

Dietary intake data were collected using a 4-day food diary, including at least one weekend day to capture variations in eating patterns. Diary days were randomly assigned by computer-assisted methods to ensure even representation across the week. Participants received detailed instructions for completing the diaries, including portion size estimation. Trained interviewers reviewed the entries with participants to verify accuracy and completeness. The verified data were coded by professionals to ensure consistency and accuracy across the dataset. To calculate mean daily consumption per individual, the 4 days were averaged.

### Classification of whole grains and quantifying consumption

Currently, WG are not formally quantified in the NDNS. To classify WG consumption, a comprehensive list of foods containing WG was compiled from the NDNS nutrient database (*n* 231 unique foods). No threshold in terms of amount of WG per serving or % of WG weight was applied. To quantify WG content and therefore consumption, the WG content of foods in grams or percent was examined using ingredient information obtained from publicly available sources such as retailer websites, brand websites, Mintel and Brand Bank. Dry matter data, commonly provided on product labels, were used to maintain consistency (e.g. oat granola 66 % WG and HOVIS wholemeal bread 62 % WG). To account for product reformulations and compositional changes over the 11-year study period, we utilised the most current publicly available data. This could lead to misspecification of WG content if a food did undergo re-formulation, though most foods in the database would not be subject to reformulation as they already contained > 90 % of dry weight from WG or would inherently be defined as 100 % of grain from whole grain (e.g. wholemeal bread, brown rice, oats or wholewheat pasta). In cases where packaging information was unavailable, often due to product discontinuation, similar products were used as substitutes. If different WG values were found for representative products, we took an average, though this occurred very infrequently. Whole grain amounts were appended to individual foods in the NDNS database. Because not many foods in the UK contain whole grains, a small number of foods account for a bulk of whole grain consumed (e.g. wholemeal bread accounted for approximately 8·5 % of total whole grains), and extra care was taken in the coding of these items. The coding process involved two independent coders with considerable experience in the analysis of population-based dietary survey data, with subsequent data review and verification to ensure accuracy.

### Dietary variables

The study examined mean WG consumption overall and by socio-demographic factors. Secondary trend analyses considered median intakes and the percent of the population consuming no whole grains, in addition, we examined whether nutrient and food group intakes differed across tertiles of WG intake.

### Covariate analysis

The analysis included several covariates: sex, age, race, income, education, BMI, cigarette smoking status and self-reported health status. Age was categorised into five groups: 1·5–3 years, 4–10 years, 11–18 years, 19–64 years and ≥ 65 years. Education level was categorised based on the age at which formal education was completed: low (≤ 16 years), moderate (17–18 years) and high (≥ 19 years), and analysis was restricted to adults. Household income was adjusted for equivalence using a score based on the household composition, and the total income was divided to create income tertiles.

Adult BMI was categorised into underweight (< 18·5 kg/m^2^), healthy (18·5–24·9 kg/m^2^), overweight (25–29·9 kg/m^2^) and obese (≥ 30 kg/m^2^). Cigarette smoking status was classified into never, former and current. Self-reported health status was categorised into three groups: very good, good and fair/poor.

**Figure 1.**
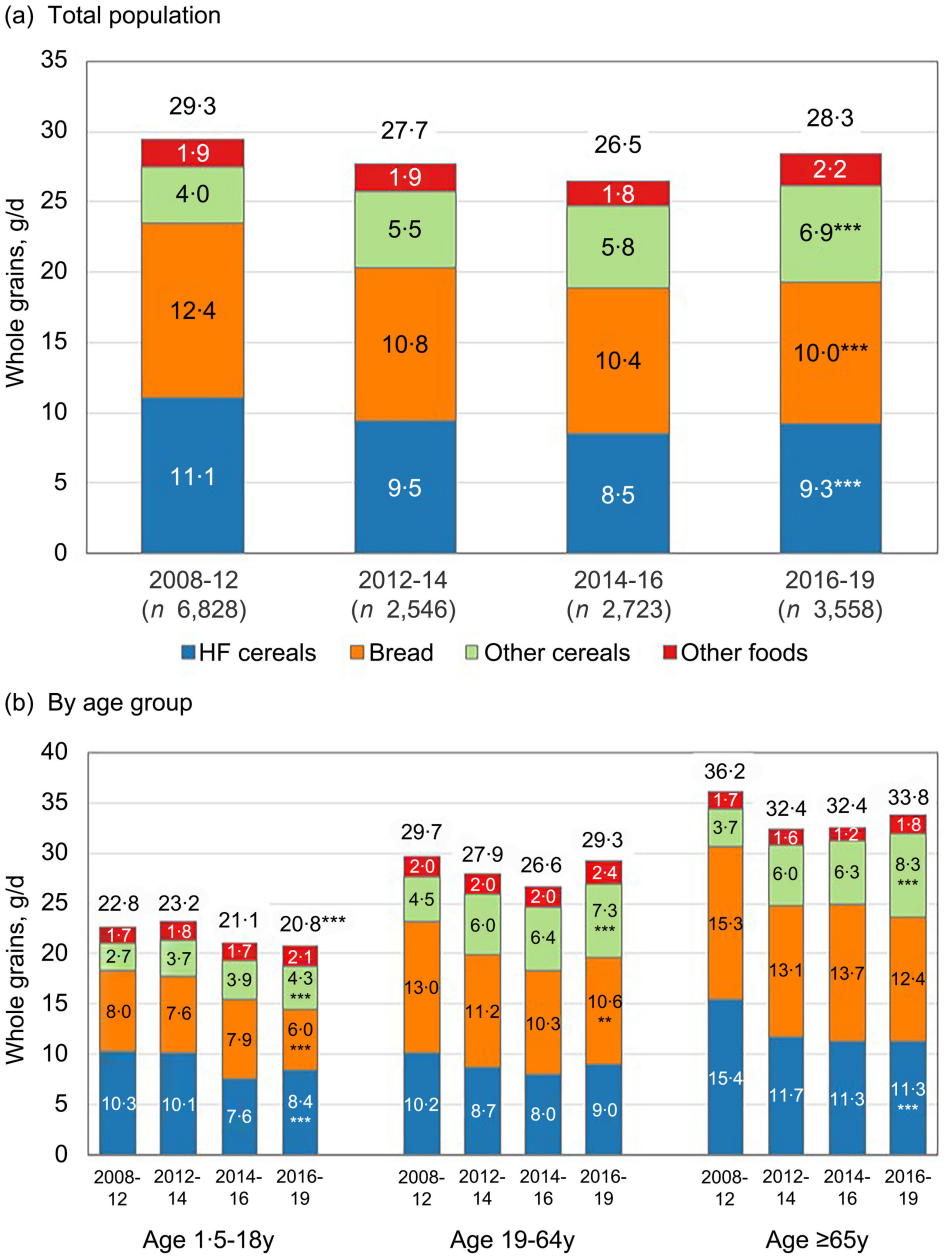
Trends in whole grains intake in the UK, overall and by source. Asterisks indicate statistical significance of *P* value for trend: *** < 0·001; ** 0·001 < *P* < 0·01; * 0·01 < *P* < 0·05. If no asterisks is shown the *P* value for trend was > 0·05 and not considered statistically significant.

The study examined mean WG consumption by socio-demographic characteristics, including age, sex, race, education and income. Additionally, trends in mean WG consumption from 2008 to 2019 were analysed, both overall and stratified by age. The analysis also included mean WG consumption by BMI, self-reported health status and smoking status. For analyses of recent socio-demographic patterns in intake, data from 2016 to 2019 were used. For trend analyses, multiple waves of data collection were pooled to increase the sample size and increase statistical precision. The total sample size for analysis was 15 655 individuals; year specific sample sizes are provided in Figure 1(a).

### Statistical analysis

Statistical analyses were conducted using SAS version 9.4, employing appropriate survey sampling weights, strata and commands to account for the complex survey design. An alpha-level of 0·05 was used for all statistical tests, and no adjustments were made for multiple comparisons given the descriptive nature of the study. Survey-weighted Wald tests and trend tests for categorical and ordered covariates were used to determine whether intakes of WG differ within category.

## Results

In the UK, the mean intake of WG did not significantly change from 2008–2012 (29·3 g/d) through 2016–2019 (28·3 g/d; *P*_trend_ = 0·16) (Figure 1(a)). Older adults consistently had the highest intakes of WG (more than 32 g/d), but intakes declined non-significantly over the study period (36·2–33·8 g/d; *P*-trend = 0·28). The intake of whole grains decreased significantly for children 1·5–3 years (–19 %; 22·5–18·3 g/d; *P*-trend = 0·037) and 4–10 years (–21 %; 26·3–20·8 g/d; *P*_trend_ < 0·001). No significant changes, either increase or decreases, were observed in other ages (see online Supplementary Table 1 for additional data by age group).

Five foods accounted for about 32 % of whole grain reports in the NDNS database: Weetabix and similar, wholemeal bread, toasted wholemeal bread, cooked oats and 50:50 white/wholemeal bread. The most consumed WG foods in this dataset included high-fibre cereals, bread and other cereals such as pasta and rice. Disaggregating trends by sources of WG (e.g. bread, high-fibre cereals, etc.) shows some interesting trends. First, intake from the two primary sources of WG decreased significantly: a 19 % reduction for bread (12·4–10 g/d) and a 16 % reduction for high-fibre cereals (11·1–9·3 g/d). In contrast, the mean consumption of other cereals (e.g. WG rice, pasta and other ready-to-eat cereals) increased from 4·0 to 6·9 g/d. (Figure 1(b)). Food category specific trends were generally consistent across population sub-groups, with the decline from high-fibre cereals particularly strong among younger children/adolescents and older adults, decreasing by 18 % (10·3–8·4 g/d) and 27 % (15·4–11·3 g/d), respectively. Additional data for smaller child/adolescent age groups are provided in online Supplementary Table 2. Downward trends in bread were comparable across all age groups. Increases for other grains were observed in all groups, being especially strong among older adults (+124 %, 3·7–8·3 g/d). Secondary analyses evaluated trends in median and inter-quartile range of intakes and the percent of the population consuming no whole grains (see online Supplementary Tables 3–4). Results were generally in-line with the analysis of the mean, except for the percentage consuming no whole grains, which decreased modestly for the overall population (from 22·0 % to 19·3 %; *P*_trend_ = 0·016), which was driven by a decrease in the adult population (from 24·4 % to 21·0 %; *P*_trend_ = 0·027). No change for this measure was seen for children/adolescents and older adults.

Table 1 shows average absolute and energy-adjusted WG intakes by key population sub-groups in the most recent period of data (2016–2019). Older adults had higher absolute intakes of WG (33·8 g/d (30·7, 36·9)), but when considering energy-adjusted intakes, a u-shaped pattern emerged, with high levels reported by young children (18·3 g/d (14·7, 22·0)) and older adults (20·6 g/d (18·9, 22·4)). Intakes were the lowest in those 11–18 years (13·0 g/d (11·7, 14·3)). Similarly, men consumed more whole grains on an absolute basis (31·4 *v*. 25·4 g/d), but upon accounting for energy, intakes did not differ (16·9 *v*. 16·8 g/d). Lower income individuals consumed significantly less WG compared with those with higher incomes (23·4 and 31·7 g/d, respectively). Current smokers had considerably lower WG intakes than both former and never smokers (16·4 g/d *v*. 33·6 and 32·4 g/d). Absolute and energy-adjusted WG intake was not associated with race, education or adult BMI category. Intakes were weakly associated with self-reported health status, but for absolute intakes only.

**Table 1.** Whole grain consumption by socio-demographic group, 2016–2019 (Mean values and 95 % confidence intervals)

	*n*	Whole grains, g/d		*P* value^*^	Energy adjusted whole grains, g/d per 1000 kcal		*P* value^*^
		Mean	95 % CI		Mean	95 % CI	
All	3558	28·2	26·8, 29·6		16·8	16·1, 17·6	
Age group				< 0·001			< 0·001
1·5–3 years	306	18·3	15·2, 21·4		18·3	14·7, 22·0	
4–10 years	725	20·8	19·1, 22·4		14·4	13·2, 15·5	
11–18 years	683	21·6	19·3, 23·9		13·0	11·7, 14·3	
19–64 years	1392	29·3	27·3, 31·3		16·5	15·4, 17·6	
65+ years	452	33·8	30·7, 36·9		20·6	18·9, 22·4	
Sex				< 0·001			0·92
Male	1636	31·4	29·1, 33·6		16·9	15·7, 18·0	
Female	1922	25·4	23·8, 27·0		16·8	15·8, 17·8	
Household income				< 0·001			0·006
Low income	1043	23·4	21·1, 25·6		15·0	13·6, 16·4	
Middle income	1015	30·0	27·0, 32·9		17·6	16·0, 19·2	
High income	1017	31·7	29·2, 34·2		18·0	16·6, 19·4	
Education				0·029			0·27
Low education	1391	26·6	24·5, 28·8		16·2	15·1, 17·4	
Middle education	780	27·8	24·7, 31·0		16·8	15·1, 18·6	
High education	1332	30·9	28·6, 33·3		17·7	16·4, 19·0	
Ethnicity				0·22			0·23
White	3118	28·7	27·3, 30·2		17·0	16·2, 17·8	
Other	433	25·7	21·1, 30·3		15·5	13·1, 17·8	
BMI category (adults)				0·34			0·86
Underweight	182	28·1	22·9, 33·3		17·4	14·2, 20·6	
Healthy weight	584	31·9	28·8, 34·9		17·9	16·2, 19·5	
Overweight	639	30·8	27·8, 33·8		17·6	16·0, 19·2	
Obese	439	28·1	24·7, 31·5		16·7	14·8, 18·7	
Tobacco use, adults				< 0·001			< 0·001
Current	312	16·4	13·6, 19·2		9·9	8·3, 11·4	
Former	443	33·6	30·0, 37·1		19·9	18·0, 21·8	
Never	1061	32·4	30·1, 34·7		18·4	17·1, 19·7	
Self-reported health, adults				0·018			0·29
Very good/excellent	608	34·0	30·6, 37·4		18·5	16·9, 20·2	
Good	801	29·3	27·0, 31·6		17·1	15·7, 18·4	
Fair/poor	432	27·0	23·3, 30·7		16·7	14·6, 18·8	

* *P* value is from a weighted Wald test indicating any heterogeneity in mean whole grain intakes for the categorical variables.

Intakes of key nutrients/food groups by tertile of WG consumption are provided in Table 2. WG intake was only weakly related to energy intake ranging from 1668 to 1781 kcal/d. Whole grain intake appeared to be positively associated with dietary fibre, potassium, Ca, Fe, Mg, fruits/vegetables, pulses/nuts and oily fish intake (*P*_trend_ < 0·001 for all). WG intake was inversely associated with free sugars, total fat, saturated fat, Na and red/processed meat intake (*P*_trend_ < 0·001 for all).

**Table 2.** Intake of key nutrients and food groups by tertile of whole grain intake, 2016–2019 (Mean values and 95 % confidence intervals)

	Tertile 1		Tertile 2		Tertile 3		*P*_for trend_
	(*n* 1184; 0–6·1 g/d)		(*n* 1188; 6·1–19·3 g/d)		(*n* 1186; 19·3+ g/d)		
	Mean	95 % CI	Mean	95 % CI	Mean	95 % CI	
Total energy (kcal)	1719	1673, 1765	1781	1737, 1827	1668	1626, 1711	0·002
Macronutrients							
Total carbohydrates, g/d	208	202, 214	216	209, 222	206	200, 211	0·06
Dietary fibre, g/d	15·0	14·6, 15·5	18·7	17·9, 19·5	21·3	20·7, 22·0	< 0·001
Free sugars, g/d	55·0	51·7, 58·4	50·0	47·5, 52·5	39·3	37·4, 41·3	< 0·001
Protein, g/d	67·9	65·9, 69·8	72·2	70·1, 74·3	70·5	68·5, 72·4	0·012
Total fat, g/d	67·0	64·9, 69·1	68·5	66·6, 70·5	62·9	60·8, 64·9	< 0·001
Saturated fat g/d	25·0	24·1, 25·8	25·6	24·7, 26·6	22·8	22·0, 23·6	< 0·001
PUFA g/d	9·4	9·0, 9·8	9·6	9·2, 9·9	9·3	8·9, 9·7	0·60
MUFA g/d	24·8	23·9, 25·6	25·2	24·5, 25·9	23·0	22·1, 23·8	< 0·001
Nutrients							
Potassium (mg)	2426	2361, 2490	2767	2695, 2839	2794	2727, 2862	< 0·001
Ca (mg)	747	723, 772	844	818, 871	853	825, 881	< 0·001
Vitamin D (µg)	4·6	3·5, 5·7	5·2	4·4, 5·9	6·4	4·8, 8·0	0·17
Fe (mg)	9·7	8·8, 10·5	11·6	10·9, 12·3	12·7	11·6, 13·8	< 0·001
Mg (mg)	220	213, 227	265	256, 274	287	278, 296	< 0·001
Na (mg)	1995	1930, 2060	1959	1900, 2018	1799	1742, 1855	< 0·001
Food groups							
Fruit and vegetables (g)	212	200, 224	278	264, 292	312	298, 327	< 0·001
Pulses and nuts (g)	16·1	14·0, 18·3	22·3	17·2, 27·4	22·9	20·2, 25·5	< 0·001
Red and processed meat (g)	49·0	45·4, 52·6	45·5	42·0, 49·0	32·0	29·7, 34·4	< 0·001
Fish (g)	16·0	14·0, 18·3	18·0	15·8, 20·2	21·8	19·6, 23·9	0·001
Oily fish (g)	6·1	4·6, 7·6	6·6	5·4, 7·9	10·0	8·7, 11·4	< 0·001

## Discussion

The findings of our study indicate that mean WG intake in the UK population remained stable, with no significant change observed in the study period. Per calorie, adults and the youngest children had the highest WG intakes. However, average WG consumption declined among younger populations, especially children aged 1·5–10 years, likely due to reduced intake of high-fibre cereals and bread.

Individuals in the highest tertile of WG consumption generally exhibit a more nutrient-dense dietary pattern compared to those in the lower tertiles. Specifically, higher WG intake was associated with higher mean consumption of fibre, potassium, Ca, Fe,Mg, fruits and vegetables and lower intake of free sugars, Na and red and processed meat. The inverse relationship between WG intake and the consumption of less healthy food groups, such as red and processed meats, and smoking habits suggests that higher WG intake is associated with more health-conscious choices.

The decrease in mean high-fibre cereal and bread consumption (traditionally the main sources of WG) highlights a potential shift in dietary patterns. Bread, a key staple in the UK diet for centuries, has seen a decline in consumption over the past 50 years^(19)^. This reduction in bread consumption may be attributed to the growing popularity of other carbohydrates and to misconceptions about bread’s role in weight gain and digestive issues, despite limited scientific evidence supporting such concerns^(19)^.

The average daily UK’s WG consumption is less than half of some European recommended levels, highlighting a substantial gap between current intake and optimal dietary targets. In the UK, there is no official definition or quantitative intake recommendation for WG, although the EatWell Guide suggests choosing higher-fibre or wholegrain varieties such as wholewheat pasta or brown rice^(17)^. European countries have varied recommendations for WG consumption. For example, the Swedish National Food Agency suggests an intake of 75 grams per 10 MJ of energy, translating to about 70 grams per day for women and 90 grams per day for men. The Norwegian Directorate of Health recommends 70–90 grams of WG per day, while Danish dietary guidelines suggest at least 75 g/d. In Europe, no single unified recommendation exists, though European Union guidelines generally encourage the consumption of WG cereals. Additionally, in the USA, guidelines recommend that at least half of all grains consumed should be WG, equating to ≥ 48–85 g/d depending on age and sex^(20)^. The Global Burden of Disease Study has established an optimal WG intake of 125 g/d (with an optimal range of 100–150 g/d), but this is intended to be used for statistical modelling as opposed to a consumer-facing recommendation^(21)^.

The analysis of U.S. WG consumption trends from 1994 to 2018^(22)^ shows that despite various dietary guidelines and policy measures, WG intake only modestly increased. While adults’ WG consumption density saw little change, a significant rise occurred among children, especially following the introduction of updated school meal standards in 2013. By 2018, school food became the leading source of WG for children, with almost half of those eating school meals consuming WG.

Consumer behaviour in dietary choices is complex and influenced by different factors, including sensory and perceptual features of the food itself, external influences like information and social dynamics as well as individual factors such as personal habits and prior experiences. Additionally, cognitive elements, including knowledge, preferences and anticipated outcomes, play a role, alongside broader sociocultural influences^(23)^. In promoting healthier dietary choices, perceived benefits play a critical role, as exemplified by dietary fibre, which supports digestive health with noticeable effect^(24)^. In contrast, whole grain consumption is more challenging to promote because its health benefits, such as reduced risks of all-cause mortality, type 2 diabetes, CVD and certain cancers, are long term and not immediately apparent. This lack of tangible feedback makes it harder for consumers to prioritise WG. Also confusion about how to locate, identify and use WG further complicates adoption, with some consumers finding it challenging to differentiate whole from refined grains^(25)^. However, simplified labelling and targeted education campaigns could reduce misunderstandings and better convey the value of WG.

Taste is another key factor influencing the consumption of WG^(25)^. Some consumers find WG products less appealing than refined alternatives due to their denser texture and stronger flavours. This taste perception can deter individuals from incorporating WG into their diets, even when they recognise the associated health benefits. Efforts to improve the palatability of whole grains can play a role in increasing WG acceptance. Strategic product development that integrates clear messaging, improved taste and accessibility alongside education and cultural considerations is essential for promoting WG consumption^(26)^. Addressing these interconnected factors can align consumer preferences with public health goals, fostering healthier dietary practices.

The recommended levels of dietary whole grain intake in Europe and the USA, while ambitious, are achievable. Evidence suggests that effective public health campaigns and strategic product development can increase WG consumption. A study^(15)^ examined the success of the Danish Whole Grain Partnership (DWP), which developed a shared knowledge base including the definition of WG, a review of health benefits, and studies on consumer knowledge and perceptions. The DWP led to several key initiatives, including revised dietary guidelines, public information campaigns, the introduction of a standardized WG logo and an increased supply of WG products. As a result, WG intake in Denmark rose by 128 % (from 36 to 86 g/MJ), and the proportion of the population meeting daily WG recommendations increased from 6 % to 54 % by 2019. The DWP’s success was attributed to factors such as strong public–private collaboration, cultural resonance with WG (e.g. rye bread) and the involvement of governmental food authorities. However, replicating this model in the UK may be challenging due to the absence of some key success factors, such as national government backing, clear WG definitions^(12)^, regulation and specific recommendations.

### Strengths, weaknesses and limitations

One of the primary challenges in dietary studies, including this one, is the risk of misreporting food consumption. To mitigate this, NDNS implemented a 4-day dietary recording period and conducted follow-up visits with participants, ensuring more accurate data collection. This method may result in some changes in reported diets as data are collected in near real time and could result in systematic under-reporting of some foods and may lead to downwardly biased estimates of total energy intake^(27)^. This is in-line with the data we observed, where energy appears to be under-reported as the energy reported does not align with the weight status of the population. However, these data remain the only source of population-based dietary data in the UK and are routinely used by NGOs and government to inform dietary recommendations and policy. For consistency, we relied on dry matter data, as it is the most commonly provided on product labels. While significant effort was made to accurately determine the whole grain content of foods, some limitations in the calculation process were likely unavoidable. For instance, when packaging information was unavailable, mostly due to product discontinuation, similar products were matched, which may have introduced minor inaccuracies for the earlier period of the analysis. The data used in this study spanned an 11-year timeframe, during which food products were subject to reformulation or changes in composition. To address this, we utilised the most up-to-date and accessible information from public resources. Despite these challenges, the study’s comprehensive approach provides a robust estimate of whole grain intake.

### Conclusion

There has been no statistically significant change in mean WG consumption in the UK between 2008/2012 and 2016/2019. UK whole grain intakes remain below the levels recommended by many other countries.

## Supporting information

Kutepova et al. supplementary material Kutepova et al. supplementary material

## Supplementary material

For supplementary material/s referred to in this article, please visit https://doi.org/10.1017/S0007114525104091
