# Supplementary material for: Whole grain intake remains unchanged in the UK, 2008/2012–2016/2019
Source: Br J Nutr. 2025 Jul 28;134(3):213–9. doi: 10.1017/S0007114525104091 (PMC12492063; doi:10.1017/S0007114525104091)
Supplement: Kutepova et al. supplementary material [file S0007114525104091sup001.docx]

**Title:** Whole Grain Intake Remains Unchanged in the United Kingdom, 2008/2012 – 2016/2019

**Authors**: Inga Kutepova, PhD^1^; Colin D Rehm, PhD^2^; Samara Joy Smith, PhD, MDiv^2^

**Supplemental Table 1.** Survey-weighted mean whole grain intakes by NDNS wave for detailed child/adolescent age groups

|  | Mean whole grains intake, g/d (95% CI) | | |  |
| --- | --- | --- | --- | --- |
|  | 1.5-3y | 4-10y | 11-18y | |
| NDNS Waves |  |  |  | |
| 2008-12 | 22.5 (20.0, 25.0) | 26.4 (24.4, 28.3) | 19.9 (18.2, 21.7) | |
| 2012-14 | 18.7 (15.7, 21.7) | 24.5 (22.2, 26.8) | 23.5 (19.5, 27.5) | |
| 2014-16 | 19.2 (16.3, 22.1) | 24.1 (21.7, 26.5) | 18.9 (16.4, 21.4) | |
| 2016-19 | 18.3 (15.2, 21.4) | 20.8 (19.1, 22.4) | 21.6 (19.3, 23.9) | |
| p-value for linear trend | ***0.037*** | ***<0.001*** | 0.57 | |
|  |  |  |  | |

**Supplemental Table 2.** Survey-weighted mean whole grain intakes by food group by NDNS wave for detailed child/adolescent age groups

|  | Mean whole grains intake, g/d (95% CI) | | |  | |  |
| --- | --- | --- | --- | --- | --- | --- |
|  | HF cereals | Bread | Other cereals | | Other foods | |
| NDNS Waves, age 1.5-3y |  |  |  | |  | |
| 2008-12 | 12.8 (10.9, 14.7) | 7.2 (5.9, 8.4) | 1.7 (1.0, 2.4) | | 0.8 (0.6, 1.0) | |
| 2012-14 | 9.8 (7.5, 12.2) | 4.9 (3.5, 6.3) | 2.8 (1.6, 4.0) | | 1.2 (0.7, 1.7) | |
| 2014-16 | 8.3 (6.2, 10.4) | 7.1 (5.2, 9.0) | 2.6 (1.8, 3.3) | | 1.2 (0.8, 1.7) | |
| 2016-19 | 8.9 (6.0, 11.8) | 4.6 (3.6, 5.6) | 3.4 (2.4, 4.5) | | 1.4 (0.9, 1.8) | |
| p-value for linear trend | ***0.012*** | ***0.016*** | ***0.010*** | | ***0.024*** | |
|  |  |  |  | |  | |
| NDNS Waves, age 4-10y |  |  |  | |  | |
| 2008-12 | 12.8 (11.4, 14.3) | 8.9 (7.8, 10.0) | 2.8 (2.3, 3.3) | | 1.8 (1.5, 2.2) | |
| 2012-14 | 11 (9.5, 12.5) | 8.0 (6.6, 9.3) | 3.4 (2.3, 4.5) | | 2.1 (1.6, 2.7) | |
| 2014-16 | 9.2 (7.8, 10.7) | 8.0 (6.6, 9.4) | 5.3 (3.8, 6.9) | | 1.6 (0.9, 2.2) | |
| 2016-19 | 9.4 (8.2, 10.5) | 5.8 (4.8, 6.7) | 3.6 (2.8, 4.3) | | 2.1 (1.6, 2.5) | |
| p-value for linear trend | ***<0.001*** | ***<0.001*** | ***0.01*** | | 0.67 | |
|  |  |  |  | |  | |
| NDNS Waves, age 11-18y |  |  |  | |  | |
| 2008-12 | 7.5 (6.6, 8.5) | 7.6 (6.5, 8.6) | 2.9 (2.2, 3.6) | | 2.0 (1.6, 2.4) | |
| 2012-14 | 9.5 (7.7, 11.3) | 8.1 (6.5, 9.6) | 4.2 (1.7, 6.8) | | 1.7 (1.2, 2.2) | |
| 2014-16 | 5.8 (4.6, 7.0) | 8.0 (6.1, 9.9) | 3.1 (2.4, 3.8) | | 2.0 (1.4, 2.6) | |
| 2016-19 | 7.3 (6.0, 8.7) | 6.7 (5.4, 8) | 5.2 (4.1, 6.4) | | 2.3 (1.8, 2.9) | |
| p-value for linear trend | 0.32 | 0.41 | ***0.003*** | | 0.32 | |
|  |  |  |  | |  | |

HF = high-fibre

**Supplemental Table 3.** Weighted median whole grain intakes by NDNS wave overall and by age group

|  | Median whole grains intake, g/d (IQR) | | | |
| --- | --- | --- | --- | --- |
|  | Total | 1.5-18y | 19-64y | 65+y |
| NDNS Waves |  |  |  |  |
| 2008-12 | 20.6 (4.1, 41.7) | 15.7 (4.4, 33.0) | 20.4 (2.2, 43.1) | 27.0 (10.6, 48.6) |
| 2012-14 | 20.0 (3.1, 42.2) | 16.1 (3.8, 32.4) | 19.2 (0.0, 43.1) | 28.4 (10.2, 47.7) |
| 2014-16 | 18.6 (4.4, 39.7) | 14.7 (4.0, 30.9) | 17.1 (3.5, 39.8) | 25.7 (9.0, 47.7) |
| 2016-19 | 21.2 (5.4, 41.2) | 14.6 (3.8, 30.5) | 21.3 (4.7, 42.0) | 29.7 (11.1, 48.1) |
|  |  |  |  |  |

IQR = inter-quartile range

**Supplemental Table 4.** Weighted percent consuming no whole grains by NDNS wave overall and by age group

|  | % consuming no whole grains (95% CI) | | | |
| --- | --- | --- | --- | --- |
|  | Total | 1.5-18y | 19-64y | 65+y |
| NDNS Waves |  |  |  |  |
| 2008-12 | 22.0 (20.5, 23.6) | 20.0 (18.2, 21.9) | 24.4 (22.2, 26.7) | 15.7 (12.5, 19.4) |
| 2012-14 | 23.3 (21.0, 25.8) | 19.8 (17.3, 22.7) | 26.1 (22.7, 29.8) | 17.7 (13.1, 23.3) |
| 2014-16 | 20.9 (18.8, 23.1) | 20.3 (17.6, 23.2) | 21.9 (18.9, 25.2) | 18.1 (13.8, 23.5) |
| 2016-19 | 19.3 (17.6, 21.1) | 20.4 (18.2, 22.8) | 21.0 (18.6, 23.8) | 12.4 (9.4, 16.2) |
| *p-value for linear trend* | ***0.016*** | 0.75 | ***0.027*** | 0.27 |
|  |  |  |  |  |
